# *e*LoriCorps Immersive Body Rating Scale and *e*LoriCorps Mobile Versions: Validation to Assess Body Image Disturbances from Allocentric and Egocentric Perspectives in a Nonclinical Sample of Adolescents

**DOI:** 10.3390/jcm11051156

**Published:** 2022-02-22

**Authors:** Johana Monthuy-Blanc, Giulia Corno, Marilou Ouellet, Fadel Touré, Francisca Bourbeau, Michel Rousseau, Audrey Charette, Nicolas Moreau, Normand Roy, Vicky Drapeau, Marie-Eve Mathieu, Stéphane Bouchard

**Affiliations:** 1 GR2TCA-Loricorps-Groupe de Recherche Transdisciplinaire des Troubles du Comportement Alimentaire, Université du Québec à Trois-Rivières, 3351, Boulevard des Forges, Trois-Rivières, QC G8Z 4M3, Canada; johana.monthuy-blanc@uqtr.ca (J.M.-B.); marilou.ouellet@uqtr.ca (M.O.); fadel.toure@uqtr.ca (F.T.); francisca.bourbeau@uqtr.ca (F.B.); michel.rousseau@uqtr.ca (M.R.); audrey.charette@uqtr.ca (A.C.); nicolas.moreau@uottawa.ca (N.M.); normand.roy@umontreal.ca (N.R.); stephane.bouchard@uqo.ca (S.B.); 2 Centre de Recherche de l’Institut Universitaire en Santé Mentale de Montréal, 7401 Rue Hochelaga, Montreal, QC H1N 3M5, Canada; 3 Chaire de Recherche du Canada en Cyberpsychologie Clinique, Université du Québec en Outaouais, 283, Boul Alexandre-Taché, Gatineau, QC J8X 3X7, Canada; 4 School of Social Work, University of Ottawa, 120 University, Office 12035, Ottawa, ON K1N 6N5, Canada; 5 Groupe de Recherche Interuniversitaire sur l’Intégration Pédagogique des Technologies de l’Information et de la Communication, Université de Montréal, 90, Avenue Vincent-d’Indy, Montreal, QC H2V 2S9, Canada; 6 Département D’éducation Physique, Faculté des Sciences de L’éducation, Université Laval, Pavillon de l’Éducation Physique et des Sports, Local 2214, 2320, Rue des Bibliothèques, Quebec, QC G1V 0A6, Canada; vicky.drapeau@fse.ulaval.ca; 7 Chaire de Recherche du Canada en Activité Physique et Obésité Juvénile, Université de Montréal, Boulevard Édouard-Montpetit, Montreal, QC H3T 1J4, Canada; me.mathieu@umontreal.ca; 8 Centre de Recherche du Centre Intégré de Santé et de Services Sociaux de l’Outaouais, 20 Rue Pharand, Gatineau, QC J9Y 6H9, Canada

**Keywords:** body dissatisfaction, body distortion, virtual reality, mobile application, convergent and discriminant validity, egocentric perceptual-sensory-affective dimension of body image

## Abstract

A growing number of studies have used virtual reality (VR) for the assessment and treatment of body image disturbances (BIDs). This study, conducted in a community sample of adolescents, documents the convergent and discriminant validity between (a) the traditional paper-based Figure Rating Scale (paper-based FRS), (b) the VR-based Body Rating Scale (*e*LoriCorps-IBRS 1.1), and (c) the mobile app-based Body Rating Scale (*e*LoriCorps-IBRS 1.1-Mobile). A total of 93 adolescents (14 to 18 years old) participated in the study. Body dissatisfaction and body distortion were assessed through the paper-based FRS, the *e*LoriCorps-IBRS 1.1 and the *e*LoriCorps-IBRS 1.1-Mobile. Eating disorder symptoms, body image avoidance, and social physique anxiety were also measured. Correlation analyses were performed. Overall, the results showed a good and statistically significant convergence between allocentric perspectives as measured by the paper-based FRS, the *e*LoriCorps-IBRS 1.1 and the *e*LoriCorps-IBRS 1.1-Mobile. As expected, the egocentric perspective measured in VR produced different results from the allocentric perspective, and from cognitive–attitudinal–affective dimensions of BIDs, with the exception of body distortion. These differences support the discriminant validity of the egocentric perspective of *e*LoriCorps-IBRS 1.1 and are consistent with emerging evidence, highlighting a difference between experiencing the body from an egocentric (i.e., the body as a subject) and allocentric (i.e., the body as an object) perspective. The egocentric perspective could reflect a perceptual–sensory–affective construction of BIDs, whereas allocentric measures seem to be more related to a cognitive–affective–attitudinal construction of BIDs. Moreover, the results support the validity of the *e*LoriCorps-IBRS 1.1-Mobile with promising perspectives of implementation among young populations.

## 1. Introduction

### 1.1. Body Image Disturbances

Adolescence is marked by physical changes due to puberty and to the development of identity, which can be challenging in social contexts where standards of beauty are related to thinness and muscularity [1]. Body dissatisfaction is dramatically widespread among adolescents: studies have reported that 24% to 46% of adolescent females are dissatisfied with their bodies. The percentages for adolescent males were lower, 12–26%, but still alarming [2,3,4,5]. Body image-related issues are associated with a very strong desire to lose weight and change body shape [6]. Indeed, according to Cazale, Paquette and Bernèche [7], 41% of adolescent females report being dissatisfied with their body and wanting to be thinner, while 8% report wanting to be heavier. For males, the study revealed 24% want to be thinner, and 24% want to be heavier. Body image disturbances (BIDs, mainly body dissatisfaction and body distortion) are particularly relevant among youth, and especially during adolescence. During this period, BIDs present major risk factors for the development, maintenance and relapse of eating disorders (EDs) [8,9], such as anorexia nervosa, bulimia nervosa and binge eating disorder [10,11,12,13,14,15]. Some studies suggest that the level of body dissatisfaction can vary across different age groups [16,17]. However, other studies have found that body dissatisfaction seemed largely stable during a lifetime [5,18,19,20] and, considering the possible major consequences associated with BIDs, it is of paramount importance to identify and prevent BIDs, to promptly intervene before they become chronic, and to improve our understanding of the underlying factors that maintain BIDs. 

Despite countless studies on body image, the definition of this concept is still not unanimous e.g., [21,22,23]. In this paper, body image is considered to be a multidimensional construct, indicating a personal and mental representation of one’s physical appearance, which encompasses body-related cognitions, emotions, behaviors, and perceptions [24,25,26]. Body image dissatisfaction (which reflects the cognitive–affective dimension of BIDs) and body distortion (which reflects the perceptual dimension of BIDs) are two of the most-studied manifestations of BIDs [27,28,29,30]. Body image dissatisfaction refers to the extent people like or dislike the shape and/or size of their own body and whether they accept and value it [27]. Body distortion refers to a disturbance in accurately estimating one’s own body size [27], and is observed when there is a difference between actual and perceived body size. The relation between these two dimensions remains an object of scientific debate [21]. Indeed, Cash and Deagle [27], and more recently Cornelissen et al. [31], suggested that attitudinal and perceptual components might represent two distinct phenomena. Nevertheless, several studies have proposed that BIDs may be driven by distorted attitudes toward a person’s own body (i.e., body dissatisfaction), indicating that perceptual distortions could be explained solely in terms of changes in attitudinal body image [21,32,33].

### 1.2. Measures of BIDs

To prevent BIDs in adolescence, it is important to be able to assess and promptly identify these disturbances. In the last few decades, several different tools were used to measure body image [21,23,27]. BIDs have usually been assessed through self-report questionnaires, such as the “Drive for Thinness” and “Body Dissatisfaction” subscales of the Eating Disorder Inventory 2 (EDI-2) [34], the Body Shape Questionnaire (BSQ) [35], the Body Image Avoidance Questionnaire (BIAQ) [36], and the Social and Physique Anxiety Scale [37]. In clinical and research settings, it is quite common to use body size estimation tasks (from the calculation of height and weight) to evaluate body distortion and body dissatisfaction using depictive or metric methods [21]. In depictive methods, participants are asked to estimate their perceived and ideal body size using figure rating scales (traditional paper-based FRS, 3D FRS) e.g., [38] or [39], deforming mirrors [40], and by deforming the entire body using photographs or videos [21,41,42,43]. With FRS, body size dissatisfaction is calculated by subtracting the ideal body size from the perceived body size, whereas body distortion corresponds to the participant’s actual BMI minus the BMI of the perceived body size. In metric methods, participants are asked to indicate the size of different body parts (e.g., shoulders, hips, waist), for instance, with a rod, a caliper, or movable markers on a wall [44,45]. These distances of different body parts are then measured, for example, in centimeters. Thus, by depictive methods participants are asked to express their judgement on a global body appearance, whereas by metric methods participants are asked to focus on single and specific body parts [21]. Self-report questionnaires have been criticized as they often yield inconsistent and inconclusive results [23,46,47]. The alternative paper-based FRS has received criticism for employing figures that display unrealistic representations of a person’s body, the lack of ecological validity due to the exclusive use of frontal displays (i.e., allocentric perspective), and the absence of figures that represent obesity [48]. To counter these limitations, a growing number of studies have recently explored the potential of virtual reality (VR) technologies by presenting scenarios through allocentric and egocentric (i.e., first person) perspectives [47,49,50]. The real-time rendering and exploration of the 3D images led to referring to virtual bodies, as opposed to only body figures, when describing the stimuli. VR allows assessment of BIDs from the same perspective as the paper-based FRS, which entails looking at bodies perceived as being presented in front of the person the allocentric perspective—(i.e., third-person point of view), and from a novel point of view, which involves looking at the body through one’s eyes as if it is experienced as the person’s own body—the egocentric perspective—(i.e., first-person point of view). Recently, researchers and clinicians started to investigate the nature and role of egocentric and allocentric body image perspectives. Indeed, Riva and Gaudio [51] proposed the Allocentric Lock Theory, which claims that: (a) the spatial allocentric perspective involves somatorepresentations (representations of the memory of the body and knowledge, beliefs, and attitudes about one’s own body); (b) the spatial egocentric perspective involves somatoperceptions (perceptions of the actual state of the body and tactile stimuli from sensory inputs); and (c) individuals with—or at risk of developing—eating disorders are “locked” into a negative allocentric memory of their own body that is not adequately corrected by the information originating from the egocentric perspective [51,52,53,54,55,56]. Concretely, this manifests itself in an individual as a disruption in the way the body is experienced and remembered, and all the sensory information stored in short-term memory that could negate this disruption (e.g., significant weight loss) cannot change the allocentric (long-term memory) body perception that remains ingrained with rigidity. As a result, the maintenance of this misperception of the body is the result of the inability to update perceptual data in long-term memory [57]. 

### 1.3. eLoriCorps-IBRS a VR-Based BIDs Assessment Tool 

In 1998, Riva and colleagues recreated, for the first time, a traditional paper-based FRS in VR. They developed the body image virtual reality scale (BIVRS) [58], which consisted of seven, and later nine, female and male virtual bodies ranging from underweight to overweight, displayed in an allocentric perspective. Since then, researchers have developed more realistic and inclusive versions of VR-based FRS—e.g., [46]. Traditionally, participants are asked to observe a line-up of 3D virtual bodies presented in an allocentric perspective and to select the body that corresponds to their perceived body size and their ideal body size. Recently, Monthuy-Blanc et al. [47] developed and documented the validity of the *e*LoriCorps Immersive Body Rating Scale version 1.0 (*e*LoriCorps-IBRS 1.0). In this first version, users could observe seven virtual bodies matching their self-reported sex, ranging from underweight to overweight (i.e., BMI from 15.00 to 33.00 kg/m^2^), presented both in an allocentric and egocentric perspective. As depicted in the article, *e*LoriCorps-IBRS 1.0 replicated the exact same position as the paper-based FRS: virtual bodies have shoulders, arms and legs slightly rotated sideways. For each perspective, participants were asked to select their ideal and perceived body. The authors found a convergent validity of the allocentric ratings of the *e*LoriCorps-IBRS 1.0 with the paper-based FRS. Results from the egocentric perspective revealed novel reflections about the nature of body image. Indeed, ideal body size and body dissatisfaction in the egocentric perspective differed when assessed by the allocentric VR-based versus paper-based FRS. However, the validity of the allocentric ratings from *e*LoriCorps-IBRS 1.0 was investigated among the adult population. Moreover, the egocentric perspective has not yet been studied in adolescents.

Displaying the virtual bodies in the same position as the body figures in the paper-based FRS represented an attempt to limit the differences between the stimuli presented on paper and in VR. However, issues arose when experiencing the virtual bodies in the egocentric perspective. When immersed in VR looking down, participants saw their body as slightly turned to the right, with a prominent left shoulder and a body position that did not match their proprioception. The attempt to keep paper and VR versions similar for measuring body image may be impractical, as there are also other small differences, such as skin textures, details of the body and so forth. With the advent of future VR applications that could provide more body sizes than the seven original paper-based FRS, the possibility to select skin tone and texture as well as hair features that match those of the users, and tailor selected features of portions of the virtual bodies to patients’ needs, the *e*LoriCorps-IBRS 1.0 was slightly revised—*e*LoriCorps-IBRS version 1.1 (*e*LoriCorps-IBRS 1.1) —to position the virtual bodies as fully facing the users in the standard anatomical position with no body rotation. Given the growing need to assess adolescents’ BIDs outside of the experimental research laboratory (e.g., ecological momentary assessment) with portable smartphone apps, an allocentric version of the virtual bodies was developed, the *e*LoriCorps mobile application (*e*LoriCorps-IBRS 1.1-Mobile) to be validated specifically with adolescents. 

### 1.4. Objectives 

The current study bears two objectives. The first objective (O.1) is to assess the convergent validity between the allocentric perspective of BIDs measured with the paper-based FRS and *e*LoriCorps (-IBRS 1.1 and -IBRS 1.1-Mobile) in an adolescent sample. The convergent validity was expected to be high between the allocentric (paper, VR, mobile application) assessments of body dissatisfaction and body distortion. The second objective (O.2) is to test the discriminant validity between the egocentric perspective of BIDs measured with the VR-based *e*LoriCorps-IBRS 1.1 and the allocentric perspective measured with paper-based FRS and *e*LoriCorps (-IBRS 1.1 and -IBRS 1.1-Mobile) in an adolescent sample. Consistently, with the adult validation study, it was expected that the egocentric VR perspective would yield results that were not strongly correlated with the allocentric (paper, VR, mobile application) measures of body dissatisfaction and perceptual body distortion [47]. Moreover, we explored the relationships between dimensions of BIDs in egocentric and allocentric perspectives and other constructs associated with BIDs, such as eating disorder symptoms (EDI-A), body image avoidance (BIAQ-A), and social physique anxiety (SPAS-12).

## 2. Materials and Methods

### 2.1. Sample 

During recruitment, 84 females (80.77%) and 20 males (19.23%), all Caucasians and Canadian residents, expressed an interest in the study. Among these 84 females, 11 presented a prior or current presence of EDs. Adolescents with missing data were removed from the database, forming a a final sample of 93 participants (72% female and 28% male). Ages ranged from 14 to 18 years (m = 15.4 s.d. 1.01). The average height, weight and BMI of the participants were, respectively, 1.66 m (s.d. 0.82), 61.1 kg (s.d. 10.85), and 22.3 kg/m^2^ (s.d. 3.94). They were recruited at schools and community organizations which hold a partnership with the research group and had previously expressed an interest in *e*LoriCorps-IBRS 1.0. Inclusion criteria required that participants were French-speaking, self-identified as female or male, and aged between 14 and 18 years.

### 2.2. Equipment and Material

The study was conducted using the *e*LoriCorps-IBRS 1.1 (see [47] for a detailed description of the first version of the VR-base scale). The virtual environment ran on an HP wx4600 PC computer (3 GHz, 3.48 GB RAM, ASUS GeForce 8800GTX graphics card; Hewlett-Packard, Montréal, QC, Canada), combined with Vuzix VR920 HMD (Vuzix, Rochester, New York, NY, USA), an InterSense Cube3 motion tracker (InterSense LLC, Billerica, MA, USA), and a hand-controlled joystick from a Wii RVL-003 (Nintendo Canada, Vancouver, BC, Canada). The VR is based on Daydream Google technology. The *e*LoriCorps mobile app runs on the Google Pixel 2 phone, Android 11 version (Octa-core 4 × 2.35 GHz Kryo and 4 × 1.9 GHz Kryo, 64 GB RAM, display AMOLED, 5.0 inches, 1080 × 1920 pixels) with a Google Daydream View headset and its Bluetooth controller. The *e*LoriCorps mobile application requires the Google View VR headset and controller. All designed by Google, MountainView, CA, USA. 

### 2.3. Assessment Measures

The sociodemographic questionnaire included height, weight (to obtain body mass index, BMI), nationality, assigned sex at birth, and age. These variables were assessed to describe the sample (see the Sample section for the statistics).

**Figure 1:**
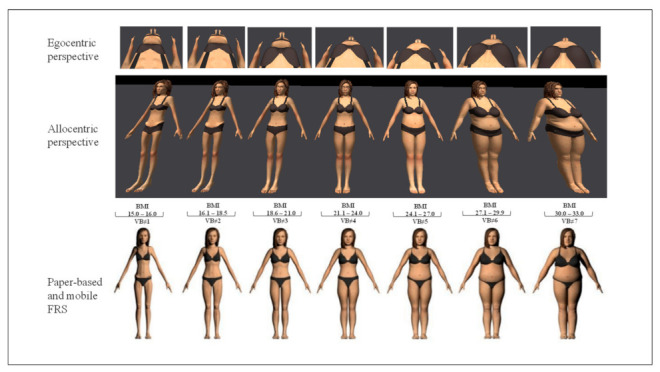
Representation of the allocentric and egocentric perspectives of *e*LoriCorps-IBRS 1.1 used with female participants.

**Figure 2:**
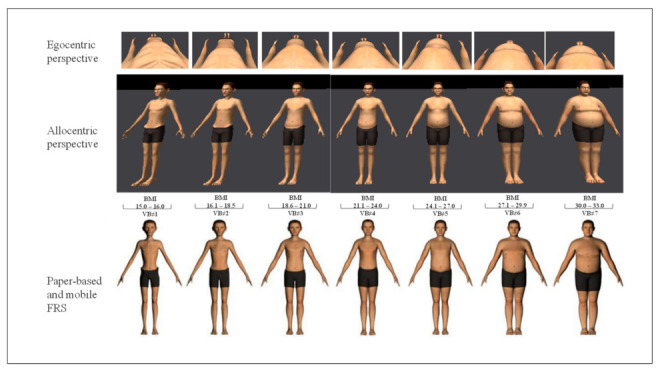
Representation of the allocentric and egocentric perspectives of *e*LoriCorps-IBRS 1.1 used with male participants.

*e*LoriCorps Immersive Body Rating Scale version 1.1 (*e*LoriCorps-IBRS 1.1). This instrument employed virtual bodies holding a standard anatomical pause see Figure 1 and Figure 2 that show screenshots from the VR-based, mobile app-based and paper-based perspective are slightly distorted compared to reality (e.g., fisheye distortion in the allocentric illustration, and variations in viewpoints in the egocentric perspective). This instrument contains two environments for assessing the allocentric and egocentric perspectives (administered in a random sequence) and a female and male version of each to be used, depending on the sex at birth of the user. A neutral VR environment was also implemented to familiarize participants with the use of the *e*LoriCorps-IBRS 1.1. In the allocentric perspective environment, participants were immersed in VR facing a line-up of seven virtual bodies and walked around each of them. After examining each virtual body for 40 to 60 seconds (see [47] for a detailed description of the procedure), the participants walked to face the virtual body that best represented their own body (Perceived Body Size—Allo. VR), and then to the virtual body they wanted to look like (Ideal Body Size—Allo. VR). In the egocentric perspective virtual environment, participants looked down at their feet and experienced each of the seven bodies for 40 to 60 seconds (see [47] for a detailed description of the procedure). Participants then transitioned to the virtual body they estimated as best representing their body size (Perceived Body Size—Ego. VR) and to the virtual body they wanted to look like (Ideal Body Size—Ego. VR).

Body Dissatisfaction scores, for the allocentric perspective (i.e., Body Dissatisfaction. VR) and the egocentric perspective (i.e., Body Dissatisfaction-Ego. VR), were calculated from the perceived body size minus the ideal body size. A positive score indicated that the participant desired a thinner body than their perceived body size, and a negative score indicated that the participant desired a larger body than their perceived body size. Scores can range from −6 (i.e., no dissatisfaction) to ± 6 (i.e., extreme dissatisfaction).

Body Distortion scores were calculated from the actual body size of the participant minus the perceived body size for the allocentric perspective (i.e., Body Distortion-Allo. VR) and the egocentric perspective (i.e., Body Distortion-Ego. VR). A positive score indicated that the participant perceived their body as thinner than their actual BMI, while a negative score meant that the participant perceived their body as bigger than their actual BMI. Scores can range from 0 (i.e., no distortion) to ± 6 (i.e., extreme distortion).

*e*LoriCorps mobile app based Body Rating Scale (*e*LoriCorps-IBRS 1.1-Mobile; developed by Loricorps’ team, at Université du Québec à Trois-Rivières, Quebec, CANADA). This instrument employed an app-based FRS with seven virtual bodies on a visual analog scale, showing a female or male version of each depending on the sex at birth of the user. A cell phone with the mobile app already open was given to the participant. Then, they were asked to observe the virtual bodies and select the one best representing their body size (Perceived Body Size—Allo. mobile) and the one they wanted to look like (Ideal Body Size—Allo. mobile). All scores range from 0 to ±6. Body Dissatisfaction scores for the mobile app (i.e., Body Dissatisfaction—Allo. mobile) were calculated from the perceived body size minus the ideal body size. Body Distortion scores were calculated for the mobile app (i.e., Body Distortion—Allo. mobile) from the actual BMI of the participant minus the BMI of the perceived body size. Scores can range from 0 (i.e., not dissatisfied) to ±6 (i.e., extremely dissatisfied).

Figure rating scale. The FRS [59] is a paper-based questionnaire consisting of seven body figures, presented in an allocentric perspective, that increase in size from thinnest to largest, numbered from 1 to 7. Participants were asked to circle their Perceived Body size and their Ideal Body size. Body Dissatisfaction (i.e., Body Dissatisfaction—Paper-based FRS) and Body Distortion (i.e., Perceptual Body Distortion—Paper-based FRS) scores were calculated as recommended by the authors [59], which is similar to the procedures employed for the *e*LoriCorps-IBRS 1.1.

Eating Disorder Inventory. The French very short version of the Eating Disorder Inventory-Adolescent version (EDI-A) represents a 16-item multidimensional self-report questionnaire that assesses symptoms of eating disorders in adolescent populations [60]. This instrument was validated among a community sample of female and male adolescents [60]. The questionnaire comprises eight subscales (i.e., Drive for Thinness, Bulimia, Body Dissatisfaction corresponding to the ED-Symptom Index as well as Ineffectiveness, Perfectionism, Interpersonal Distrust, Interoceptive Awareness and Maturity Fears corresponding to the ED-Personality-Trait Index) and is based on a Likert scale from 0 “not at all” to 5 “extremely.” The EDI total score, index score and each subscale total scores were reported. In our sample, Cronbach’s alpha was 0.78, showing excellent internal consistency [61].

Social Physique Anxiety Scale. The Social Physique Anxiety Scale (SPAS-12) [37], validated in French by Maïano et al. [62], is a 12-item self-report scale developed to assess the degree to which people become anxious when others observe or evaluate their physiques. This questionnaire was validated among a community sample of male and female adolescents [62]. The instrument is based on a Likert scale from 1 “not at all” to 5 “extremely”. In our sample, Cronbach’s alpha was 0.92, illustrating excellent internal consistency [61].

Body Image Avoidance Questionnaire. The Body Image Avoidance Questionnaire (BIAQ-A) [36], Adolescents French version of Maïano et al. [63], is a 19-item self-report measure of behavioral avoidance of situations and experiences that could provoke concerns about one’s own physical appearance, such as social activities that involve eating or wearing tight-fitting clothes. The questionnaire was validated among a community sample of female and male adolescents [63] and is characterized by four subscales: clothing (i.e., wearing clothes that hide one’s own body), social activities (i.e., avoiding social activities that imply eating or that draw attention to one’s own body), eating restraint, and grooming/weighing (i.e., checking behaviors such as weighing or scrutinizing one’s own body in the mirror). Items can score from 0 “never” to 5 “always”. The questionnaire’s internal consistency reliability (in the present study: Cronbach α = 0.63) is consistent with what was reported in other studies (e.g., Cronbach α ranged between 0.64 and 0.89 [64]).

Simulator Sickness Questionnaire. The French version of the Simulator Sickness Questionnaire [65] measures cybersickness or the presence of physiological discomfort during VR immersion. The 16-item questionnaire employs a Likert scale from 0 “none” to 3 “severe”. In our sample, Cronbach’s alpha was 0.84, indicating excellent internal consistency [61]. SSQ total raw scores were calculated, as recommended by Bouchard et al. [66].

### 2.4. Procedure

The study protocol was approved beforehand by the ethics committees of the Université du Québec à Trois-Rivières (UQTR; CER-21-280-08-02.24). Parents were informed of the study but their consent was not required. In Quebec, the participation of teenagers aged 14 and over does not require parental consent. The study did not include any compensation since it was integrated into the community’s regular activities. Participants were recruited from 285 schools in Quebec. Students aged 14 and older were informed about the study directly in their classes, by the teacher, or during FitSpirit physical activity (visit the website www.fitspirit.ca for further information; Leduc et al. [67]). They were free to participate or not and were required to speak with the project contact representative of their school or FitSpirit-referent-person if they expressed interest in participating. The research assistants responsible for collecting the data in schools were Ph.D. students. The height and weight of the participants were measured without shoes. Then, all participants completed the paper-based questionnaires (EDI-A, BIAQ, SPAS) and the paper-based FRS. Next, the *e*LoriCorps-IBRS 1.1 and *e*LoriCorps-IBRS 1.1-Mobile versions were administered, randomly, by the experimenter. Before administering the test, the experimenter explained the procedure to participants and provided them with bottled water (in case they felt ill). Participants were informed that some cybersickness could occur and were encouraged to mention it if it happened. After the experiment with the *e*LoriCorps-IBRS 1.1, the Simulator Sickness Questionnaire was administered to all participants. Finally, the participants were invited to document their immersive experience through four open questions related to their impressions and feelings towards the immersions.

### 2.5. Statistical Analysis

In comprehensive and comparative perspectives, the data analysis is based on virtual body score for (i) actual body size and (ii) perceived and ideal body size measures. For the actual body size measure, see Figure 1 and Figure 2 for the correspondence between VB and BMI. For perceived and ideal body size measures, virtual body-VB#1 corresponds to visual analog scale (VAS) less than or equal to 14%; virtual body VB#2 corresponds to VAS between over 14% and equal to or less than 29%; virtual body VB#3 corresponds to VAS between over 29% and equal to or less than 43%; virtual body VB#4 corresponds to VAS between over 43% and equal to or less than 57%: virtual body VB#5 corresponds to VAS over 57% and equal to or less than 71%; virtual body VB#6 corresponds to VAS over 71% and equal to or less than 86%; virtual body VB#7 corresponds to VAS over 86%. To document the potential impact of the participant’s sex at birth on the results, all statistical analyses were also performed separately for females and males. The results did not differ when analyzed separately for each sex (i.e., significant differences remained significant, and non-significant differences remained non-significant). Therefore, to maximize statistical power, results for the aggregated sample were reported (results analyzed by sex are available upon request). Parametric variables were represented as mean ± standard deviation (SD). The statistical analysis was performed using Stata 16.1 software. Pearson correlation was performed to analyze the relationship between questionnaires and body dissatisfaction and body distortion. Since multiple analyses were performed in this study, a correction for the inflation of Type 1 error was applied to the alpha level to consider results of objectives 1 (O.1) and 2 (O.2) as statistically significant. A Bonferroni correction was applied familywise for each component of BIDs (i.e., for Perceived Body Size score, Ideal Body Size score, Body Dissatisfaction score, and Body Distortion score), resulting in a *p*-value lower than 0.008 (0.05/6) for Pearson correlations. Regarding BMI, two outliers were found in the data. Since raw BMI data were not used in the analysis (only used to calculate the body distortion), the potential effect of the two outliers on the results became neutralized. Other data were normally distributed. An a priori power analysis was conducted using G*Power3 using a two-tailed test, a medium effect size (*d*= 0.50), and an alpha of 0.05. Result showed that a total sample of 29 participants was required to achieve a power of 0.80 [68].

## 3. Results

Descriptive statistics on the main measures are reported in Table 1.

Analyses for the convergent and discriminant validity are reported in Table 2. Results revealed almost all correlations were statistically significant, and the application of a strict Bonferroni correction did not strongly influence the findings. The significance level must also be interpreted in the context of a large sample, hence focusing more on the strength of the correlations than on the significance levels.

The convergent validity of the *e*LoriCorps-IBRS 1.1-Mobile and the FRS allocentric perspective assessments of body image (O.1) was good (mean *r* = 0.71, *ps* < 0.000) for perceived body size and body distortion. Correlations were still significant, but lower (mean *r* = 0.53, *ps* < 0.000) for ideal body size and for body dissatisfaction. Correlations between the allocentric VR and mobile versions of the *e*LoriCorps-IBRS 1.1 were strong for perceived body size estimation and body distortion (mean *r* = 0.73, *ps* < 0.000), and lower for ideal body size estimation and body dissatisfaction (mean *r* = 0.49, *ps* < 0.000), and thus consistent with patterns observed with the FRS. 

The discriminant validity of the egocentric perspective from the *e*LoriCorps-IBRS 1.1 (O.2) in this adolescent sample was supported by low correlations between assessments from the allocentric perspective of the paper based-FRS vs. the egocentric perspective of the *e*LoriCorps-IBRS 1.1 for perceived body size (*r* = 0.45, *p* < 0.008), and body dissatisfaction (*r* = 0.46, *p* < 0.008), and by a non-significant correlation regarding the assessment of ideal body size (*p* = 0.06). Contrary to expectations, a higher correlation was identified for the assessment of body distortion (*r* = 0.76, *p* < 0.008). The same pattern of results was discovered for the correlations between the allocentric assessments vs. the egocentric ones of the *e*LoriCorps-IBRS 1.1, except for body distortion (*r* = 0.82, *p* < 0.008). Concerning the allocentric perspective of *e*LoriCorps-IBRS 1.1-Mobile, results showed lowest correlations with the egocentric perspective of the *e*LoriCorps-IBRS 1.1 for perceived body size (*r* = 0.31, *p* = 0.003), and by a non-significant correlation regarding the assessment of ideal body size (*p* = 0.44), and body dissatisfaction (*p* = 0.025). As previously mentioned, a higher correlation was identified for the assessment of body distortion (*r* = 0.67, *p* < 0.008). To show that correlations in favor of discriminant validity are indeed different from those in favor of convergent validity, correlation coefficients were compared with document statistical differences (Field, 2018). Correlations between the egocentric VR-based perspective and the allocentric VR-based perspective were significantly lower than the correlations between the allocentric-VR based perspective and the allocentric paper-based perspective for perceived body size (*p* < 0.01), ideal body size (0.02 < *p* < 0.05) and body dissatisfaction (*p* < 0.01). Regarding body distortion, the correlation between the egocentric VR-based perspective and the allocentric VR-based perspective was significantly higher than the correlation between the allocentric-VR based perspective and the allocentric paper-based perspective (0.02 < *p* < 0.05). Correlations between the egocentric VR-based perspective and the allocentric paper-based FRS were significantly lower (*ps* < 0.01) than correlations between the allocentric VR-based perspective and the allocentric paper-based FRS for perceived body size, ideal body size and body dissatisfaction. The correlation between the egocentric VR-based perspective and the allocentric paper-based FRS was not significantly different (*p* > 0.20) from the correlation between the allocentric VR-based and the allocentric paper-based FRS for body distortion. Correlations between the egocentric VR-based and the allocentric mobile-based perspectives were significantly lower (*ps* < 0.01) than correlations between the allocentric VR-based and the allocentric mobile-based perspectives for perceived body size, ideal body size and body dissatisfaction. The correlation between the egocentric VR-based perspective and the allocentric mobile-based perspective was not significantly different (0.10 < *p* < 0.20) from the correlation between the allocentric VR-based perspective and the allocentric mobile-based perspective for body distortion. In summary, the discriminant validity of the egocentric perspective from the *e*LoriCorps-IBRS 1.1 is particularly confirmed with the allocentric perspective of the technology-based method (*e*LoriCorps-IBRS 1.1 and *e*LoriCorps-IBRS 1.1-Mobile) to measure ideal body size.

Exploratory discriminant validity analyses display significant correlations between body dissatisfaction and body distortion scores and external variables (see Table 3 and Table 4). The allocentric *e*LoriCorps-IBRS 1.1 and *e*LoriCorps-IBRS 1.1-Mobile assessments of body dissatisfaction were significantly related to the total score of the BIAQ. Furthermore, both the mobile- and VR-based allocentric body distortion assessments were significantly correlated with the total score of the EDI-A, the Personality Index and Symptoms Index, whereas the total score of the BIAQ was significantly associated only with the VR-based allocentric body distortion assessment. The SPAS-12 was not significantly associated with body dissatisfaction or with body distortion. Body dissatisfaction and body distortion measured in the allocentric paper-based FRS were not significantly associated with any attitudinal–affective–cognitive variables associated with BIDs.

Regarding the specific correlations between the EDI-A and BIAQ subscales and body dissatisfaction and body distortion, the allocentric *e*LoriCorps-IBRS 1.1 body dissatisfaction assessment was significantly correlated to maturity fear and eating restraint, whereas the allocentric *e*LoriCorps-IBRS 1.1-Mobile body dissatisfaction assessment was significantly associated with maturity fear, covering up the body with clothes that hide one’s own body, and avoidance of social activities that could provoke concerns about one’s own physical appearance. Body dissatisfaction measured in the egocentric VR-based perspective was negatively associated with interoceptive awareness. Eating restraint significantly correlated with all measures of body distortion, whereas avoidance of social activities was significantly related to body distortion measured in the allocentric VR-based perspective. Finally, maturity fear was significantly related to body distortion, measured in the allocentric paper-based and VR-based condition and in the egocentric VR-based condition. The relationships were medium with correlations ranging from 0.21 to 0.29 [69]. These effect sizes appear to be generally lower for body distortion than for the body dissatisfaction scores.

## 4. Discussion

The current study examined (O.1) the convergent validity between the allocentric-based assessments (paper-based FRS, *e*LoriCorps-IBRS 1.1 and *e*LoriCorps-IBRS 1.1-Mobile) of BIDs and (O.2) the discriminant validity of the egocentric perspective (measured with the VR-based *e*LoriCorps-IBRS 1.1) versus the allocentric-based assessments (paper-based FRS, *e*LoriCorps-IBRS 1.1 and *e*LoriCorps-IBRS 1.1-Mobile) of BIDs in a community sample of adolescents. Moderate convergent validity was discovered between the allocentric (paper, VR, mobile application) assessments of body dissatisfaction and body distortion. As expected, the egocentric VR perspective yielded results that were not strongly correlated with the allocentric (paper, VR), and not significantly correlated with the allocentric mobile-based measures of body dissatisfaction. However, correlations did not significantly differ regarding body distortion. Concerning the exploratory discriminant analyses, the different strengths of relationships between dimensions of BIDs in egocentric and allocentric perspectives and other attitudinal–affective–cognitive variables associated with BIDs, such as eating disorders symptoms (EDI-A), body image avoidance (BIAQ-A), and social physique anxiety (SPAS-12), are further discussed in the following paragraphs. Adolescents who participated in this study reported marginal dissatisfaction about their body size. Participants tended to slightly overestimate their body size when using traditional paper-based FRS, and to slightly underestimate it when using *e*LoriCorps-IBRS 1.1-Mobile. Participants tended to select an ideal body closer to their actual body size when using the paper-based FRS than when using a technology-based instrument. The overall results of convergent and discriminant validity tended to reveal two main observations. First, it must be noted that for all body distortion measures, participants’ means were close to zero and standard deviations represented the highest of all measures. This observation is consistent with findings from other authors who stated that body distortion appears primarily in clinical populations rather than in the general population [21,70].

### 4.1. Allocentric Convergent and Egocentric Discriminant Validity of the eLoriCorps-IBRS 1.1 in Adolescents

Convergent validity (O.1) between the allocentric perspective of the *e*LoriCorps-IBRS 1.1 and the paper-based FRS was confirmed when assessing perceived body size, ideal body size, body dissatisfaction, and body distortion. These results obtained from a community sample of adolescents mirror the results that the authors found when validating the first version of the *e*LoriCorps (i.e., *e*LoriCorps-IBRS 1.0) among a community sample of adults [47], and were in line with results from Fisher et al. [71], who did not observe a significant difference in assessing body distortion between a paper-based FRS and a VR-based FRS. Convergent validity was found between the *e*LoriCorps-IBRS 1.1-Mobile and both the allocentric perspective of the *e*LoriCorps-IBRS 1.1 and of the paper-based FRS for all body image-related variables (i.e., perceived body size assessment, ideal body size assessment, body dissatisfaction, body distortion). These results are quite encouraging when researchers and clinicians work with adolescents. The use of the mobile application *e*LoriCorps-IBRS 1.1-Mobile can offer a more portable and acceptable way to assess the allocentric perspective of body image-related variables among youth populations. These mobile-based methods might be more appealing for teenagers, who already integrate cell phone usage in their daily routine to accomplish different tasks (e.g., searching for information, interacting with peers, watching videos and playing games), and particularly when implementing the cognitive (allocentric) and perceptual (egocentric) training of BIDs.

Discriminant validity (O.2) between the egocentric- and allocentric-based assessments was confirmed for all body image-related variables with the exception of body distortion. More precisely, results seem to indicate that assessing ideal body size, perceived body size and body size dissatisfaction in an egocentric perspective is somewhat different than assessing the same phenomena in an allocentric perspective, both in VR-based and mobile-based assessments and by using paper-based FRS. Similar results were obtained by the authors among a community sample of adults [47].

### 4.2. Egocentric-Perceptual-Sensory-Affective Construction: The Hidden and Deepest Part of the “BIDs Iceberg”?

Experiencing the body in an egocentric perspective might be quite different than experiencing the body from a “third-person” point of view. Indeed, the body is experienced both as an object (i.e., third-person allocentric perspective) in the physical world, and as a subject (i.e., first-person egocentric perspective), on a daily basis. This is particularly true during the “famous storming-adolescent-period” which constitutes a real challenge due to both the physical and psychological upheaval [72]. The “object-body”, theorized by Foucault (1975) as “Body-machine”, is experienced, judged, and observed by others only in an allocentric perspective, and vice versa. The allocentric representation of one’s own body is constantly compared with the allocentric perspective of others’ bodies. The internalization of an observer’s point of view on one’s own body is called “self-objectification” [73], which implies the internalization of an objectified cultural ideal of beauty. In Canadian society, the youth population (as our sample) is vulnerable to the widespread societal messaging prevalent in Western cultures, which favors thin, muscular bodies [74]. The fear to change one’s own body was highlighted by our results on the correlation between BIDs and maturity fears. A mechanism of constant body surveillance or systematic monitoring of one’s own body is triggered. The discrepancy between one’s body-as-an-object and this ideal-cultural-prescription, can provoke body dissatisfaction, body shame, social physique anxiety, body checking, and body avoidance, which are all recognized predictors for the development of eating disorders [55,75,76,77,78,79]. While the body as-an-object could be more exposed to inter-individual comparison in a (Western) cultural context, the egocentric construction of the body-as-a-subject, could be exposed to intra-individual comparison in one’s own ecological daily context (mood, physiological hunger, and satiety sensations, etc.). Indeed, as theorized by the Allocentric Lock Theory, the egocentric perspective involves somatoperceptions, more precisely interoceptive percepts about the nature and state of the body [51,52,53,54,55,56]. Thus, the perception of the body from a first-person point of view could be influenced by one’s own interoceptive awareness, which refers to the ability to identify, access, understand and respond appropriately to internal bodily signals [80]. More precisely, this subjective perception of sensations arising from inside the body encompasses the proprioceptive experience of body state, the perception of hunger/satiety signals, cardiovascular, respiratory, colorectal and urinary sensations. The results of the present study appear to point in this direction, as a significant negative correlation was identified between the egocentric assessment of body dissatisfaction and interoceptive awareness. However, future studies are required to shed light on this association. An increasing number of studies have been investigating impaired interoceptive awareness in eating disorders (composing the famous EDI measurement) [81], especially among anorexia and bulimia nervosa patients [82]. This suggests that interoceptive awareness disturbances are both a vulnerability and a reinforcing factor of a pathological drive for thinness leading to restrictive eating [34,83,84,85,86,87]. Furthermore, exploring bodily sensations from an egocentric perspective can increase one’s own ability to reconnect with internal bodily signals, especially food-related signals (hunger and satiety cues). This echoes two different intervention approaches: (i) yoga allows to bridge the gap between the mind–body experience and enhances the experience of embodiment [88], and (ii) an adaptative nutritional approach—intuitive eating [89]—to decrease the negative impacts of restrained eating [47,87,90,91]. The intuitive movement (yoga) and eating are employed in the recent prevention and treatment of eating disorders, including binge-eating disorder [92,93]. In conclusion, the egocentric perspective could reflect a perceptual–sensory–affective construction of the body, whereas an allocentric representation could reflect a cognitive–affective–attitudinal construction of the body. Therefore, egocentric- vs allocentric-based body image disturbances become two sides of the same coin.

### 4.3. Implications for Innovative Integrative Intervention

Traditionally, BIDs were assessed through different methods, such as self-reported questionnaires, depictive and metric body size estimation tasks. While body image distortion can be measured only by body size estimation tasks, body dissatisfaction can be measured by either self-reported questionnaires or body size estimation tasks. But, when we are implementing these two evaluative methods, are we sure that we are measuring the same phenomenon? In short, we do not believe so. Body dissatisfaction as measured by well-established self-reported questionnaires was not associated with the same construct assessed by any of the depictive methods used in this study. We could hypothesize that these two methods are assessing different shadows of body dissatisfaction. The self-reported questionnaire could be assessing cognitive–attitudinal–affective dimensions of body dissatisfaction [81], whereas through depictive methods it is also possible to reach and explore perceptual–sensory–affective dimensions of body dissatisfaction (i.e., the lived body) [94], and particularly the sensory dimension, owing to embodiment-egocentric perspectives, as they require judgement and evaluation of the physical dimensions of the body.

In preventive and therapeutic implications, this study supports the hypothesis of the self-objectification of the allocentric perspective of the body, which may be more related to phenomena such as avoidance of situations that could trigger concerns about one’s own physical appearance, and dysfunctional eating attitudes such as body checking and the drive to achieve thinness. Indeed, in this developmental period and social context, the adolescents’ object–body should represent the target content of an eating disorder preventive program [95]. On the other hand, the egocentric body representation could tap into something different from the allocentric perspectives via the perceptual–sensory–affective construction of the body. In the preventive view, the change from allocentric–object–body to egocentric–subject–body could be akin to the change from cultural beauty to inner beauty through the acceptance process. In this case, since the virtual bodies represent the adult body, it may be possible that adolescents of the current study can project themselves more easily in the allocentric perspective, as the representation of their body in mind is related to an interpersonal comparison, which is driven by the internalization of the ideal body. In the egocentric perspective, it may be harder for adolescents to embody the bodies of adults as the egocentric perspective is an intraindividual experience. Targeting BIDs is one of the most difficult preventive and therapeutic goals to achieve, and yet body image remains to be fully understood. Knowing that BIDs are not only related to the cognitive representation of the body in memory, but also to the body perception-driven input from multiple sensory modalities, may allow the development of innovative treatments based on cognitive and perceptual training. These integrated treatments based on holistic body experiences should include multidimensional modalities of BIDs, targeting the body-as-an-object/subject. An increasing number of VR-based interventions focus on targeting different facets of BIDs. However, to the author’s knowledge, only one study protocol [96] and a pilot study [97] proposed an intervention targeting both egocentric and allocentric BIDs. In line with recent studies focused on a comprehensive integrated model [98,99], the promising VR-based integrated interventions could address the perceptual-dual-disorders (e.g., eating disorders, body dysmorphic disorder, obsessive-compulsive disorder [100]) by taking into account the current gender-inclusive society [101].

### 4.4. Strengths and Limitations in this Current Study Inspiring Further Studies

There were several strengths to this study combining BIDs among an adolescent sample, in an egocentric–immersive–virtual perspective and an ecological non-immersive mobile application. To our knowledge, the current study constitutes the first validation of a VR-based assessment of BIDs (*e*LoriCorps-IBRS 1.1) in a community sample of adolescents with a larger sample than previous VR-based studies [102]. Consistent with the recent VR-based validation in a community sample of adults [47], the present study highlights the contribution of an egocentric–immersive–virtual perspective to understand the perceptual–sensory–affective construction of the body image (vs. allocentric–cognitive-affective–attitudinal construction). The validation of the mobile application version (*e*LoriCorps-IBRS 1.1-Mobile) implies favoring the use of mobile-based assessment methods in self-regulation (preventive intervention) and self-management (therapeutic intervention). The main limitation of this study is the lack of “gold-standard” psychometric instruments to compare with the egocentric VR perspective, which is a novel tool. Another important limitation is that the virtual bodies were more representative of adult bodies than adolescent ones. In order to maintain a certain consistency between the instruments, the adult version of the paper-based FRS was preferred to the adolescent version [103]. This limitation did not seem to have impeded participants from identifying a perceived body size that was close to their actual body size. Results of the exploratory analyses should be taken with caution as multiple correlations were performed. Finally, another limitation was the small number of male adolescents participants compared with the number of female adolescents participants. Future studies should include the same proportion of female and male adolescents in order to explore possible gender-based differences.

Future studies should examine the validity of *e*Loricorps-IBRS 1.1 in an eating disorder sample. More precisely, people with a high BMI may perceive themselves to be larger than the response scale allows, which may lead to a significant response bias, especially in clinical eating disorder populations, including all body weights and shapes. Consequently, it is possible to note that the number of available virtual bodies in the *e*Loricorps-IBRS continuum might restrict people in their choices. Moving toward a nine virtual body continuum could help limit this bias. In line with this limitation, it could prove necessary to develop and assess virtual bodies that better represent the morphological characteristics of adolescents’ bodies. Furthermore, the promising perspectives of self-management interventions that focus on cognitive and perceptual training of BIDs include the development and assessment of a mobile egocentric-based version of the *e*Loricorps-IBRS 1.1. Indeed, an allocentric and egocentric mobile assessment instrument could be particularly suitable for addressing evaluation and prevention programs within a youth population who develop object-body and subject-body simultaneously. In comprehensive research on self-objectification of the allocentric perspective of the body, the question of whether there are differences when employing adolescent and adult versions of the body continuum should be explored. Indeed, bodily experience, more precisely self-objectification, may play an important role in the development and maintenance of eating disorders [8]. 

## 5. Conclusions

In conclusion, this validation study provides evidence that the allocentric perspective of *e*LoriCorps-IBRS 1.1 is a valid tool to assess perceptual–sensory–affective dimensions (egocentric perspective) and cognitive–affective–attitudinal (allocentric perspective) dimensions of BIDs in adolescents. As expected, when comparing it with the validation of the *e*LoriCorps-IRBS 1.0 in a community sample of adults, the egocentric perspective measured in VR produced different results compared to all measures from the allocentric perspective [paper-based FRS, *e*LoriCorps (-IBRS 1.1 and -IBRS 1.1-Mobile)] and from cognitive–attitudinal–affective dimensions of BIDs (EDI-A, BIAQ-A and SPAS-12). These differences support the discriminant validity of the egocentric perspective of *e*LoriCorps-IBRS 1.1 and are consistent with emerging evidence that the egocentric perceptive could reflect a perceptual–sensory–affective construction of BIDs. Allocentric measures appear to be more related to a cognitive–affective–attitudinal construction of BIDs. Moreover, the results support the validity of rating virtual bodies using the *e*LoriCorps-IBRS 1.1-Mobile, with the potential to be more enticing and to enable ecological momentary assessment. Furthermore, it could be suitable to develop a specific-fitting adolescent virtual body continuum integrated into a mobile egocentric-based version of the *e*Loricorps-IBRS 1.1 in order to detect, prevent and treat eating disorders.

## Figures and Tables

**Table 1:** Descriptive statistics.

		M	SD	Min–Max
Actual Body Size-adj		3.84	1.34	1–7
Perceived Body Size score				
Paper-based FRS		4.12	0.87	2–6
Allo. VR		3.83	0.79	2–6
Ego. VR		3.77	0.74	2–6
Allo. Mobile		3.64	0.95	1–6
Ideal Body Size score				
Paper-based FRS		3.89	0.68	2–6
Allo. VR		3.45	0.65	1–5
Ego. VR		3.51	0.73	1–6
Allo. Mobile		3.44	0.95	1–6
Body Dissatisfaction				
Paper-based FRS		0.23	0.90	−2–+3
Allo. VR		0.38	0.75	−1–+2
Ego. VR		0.27	0.61	−1–+2
Allo. Mobile		0.17	0.85	−2–+2
Body Distortion				
Paper-based FRS		−0.28	1.07	−3–+3
Allo. VR		0.38	1.42	−3–+4
Ego. VR		0.06	1.31	−3–+4
Allo. Mobile		0.17	1.05	−2–+4
EDI-A	Total	27.30	10.53	0–60
	Symptoms Index	9.27	5.02	0–25
	Personality Trait Index	20.09	7.92	0–45
	Body Dissatisfaction	4.81	1.82	1–10
	Drive for Thinness	3.26	3.20	0–10
	Bulimia	2.05	1.98	0–9
	Ineffectiveness	4.21	1.43	2–10
	Perfectionism	4.57	2.35	0–10
	Interpersonal Distrust	5.51	2.28	0–10
	Interoceptive Awareness	5.21	1.65	0–10
	Maturity Fears	2.22	2.28	0–10
SPAS-12	Total	34.38	5.94	24–50
BIAQ-A	Total	23.61	7.98	7–43
	Clothing	11.25	4.78	2–24
	Social Activities	1.61	2.94	0–12
	Eating Restraint	3.36	2.49	0–15
	Grooming and Weighing	7.38	2.76	0–13
SSQ	Total raw score	18.88	4.13	16–38

Note. BMI: Body Mass Index; Paper-based FRS: allocentric paper-based Figure Rating Scale; Allo. VR: Allocentric perspective from the *e*LoriCorps-IBRS 1.1; Ego VR: Egocentric perspective from the *e*LoriCorps-IBRS 1.1; Allo. Mobile: Allocentric perspective from the *e*LoriCorps-IBRS 1.1-Mobile; EDI-A: Eating Disorder Inventory-Adolescent version; SPAS-12: Social Physique Anxiety Scale; BIAQ-A: Body Image Avoidance Questionnaire-Adolescent version; SSQ: Simulator Sickness Questionnaire.

**Table 2:** Convergent and discriminant validity in adolescents, assessed by Pearson correlations for each component of BIDs between the allocentric perspective (paper, virtual reality, mobile application) and egocentric perspective (virtual reality by the *e*LoriCorps-IBRS 1.1) assessments.

	Pearson Correlation	*p*-Value
Perceived Body Size score		
Paper-based FRS vs. Allo. VR	0.73	<0.008 *
Paper-based vs. Ego. VR	0.45	<0.008 *
Allo. VR vs. Ego. VR	0.49	<0.008 *
Paper-based FRS vs. Allo. Mobile	0.66	<0.008 *
Allo Mobile vs. Allo. VR	0.72	<0.008 *
Allo Mobile vs. Ego. VR	0.32	0.003 *
Ideal Body Size score		
Paper-based FRS vs. Allo. VR	0.53	<0.008 *
Paper-based vs. Ego. VR	0.20	0.06
Allo. VR vs. Ego. VR	0.29	<0.008 *
Paper-based FRS vs. Allo. Mobile	0.48	<0.008 *
Allo. Mobile vs. Allo. VR	0.40	<0.008 *
Allo. Mobile vs. Ego. VR	0.08	0.44
Body Dissatisfaction score		
Paper-based FRS vs. Allo. VR	0.68	<0.008 *
Paper-based vs. Ego. VR	0.46	<0.008 *
Allo. VR vs. Ego. VR	0.47	<0.008 *
Paper-based FRS vs. Allo. Mobile	0.58	<0.008 *
Allo. Mobile vs. Allo. VR	0.58	<0.008 *
Allo. Mobile vs. Ego. VR	0.24	0.025
Body Distortion score		
Paper-based FRS vs. Allo. VR	0.74	<0.008 *
Paper-based vs. Ego. VR	0.76	<0.008 *
Allo. VR vs. Ego. VR	0.82	<0.008 *
Paper-based FRS vs. Allo. Mobile	0.75	<0.008 *
Allo. Mobile vs. Allo. VR	0.73	<0.008 *
Allo. Mobile vs. Ego. VR	0.67	<0.008 *

Note. Paper-based FRS: allocentric paper-based Figure Rating Scale; Allo. VR: Allocentric perspective from the *e*LoriCorps-IBRS 1.1; Ego VR: Egocentric perspective from the *e*LoriCorps-IBRS 1.1; Allo. Mobile: Allocentric perspective from *e*LoriCorps-IBRS 1.1-Mobile. * *p* < 0.008.

**Table 3:** Pearson correlations (with exact *p* values in brackets) for the total of questionnaires measuring components of BID and three rating scales administered to adolescents: a paper-based FRS, the *e*LoriCorps-IBRS 1.1 allocentric and egocentric perspectives, and the *e*LoriCorps-IBRS 1.1-Mobile allocentric perspective.

	EDI-A	EDI-A-S-Index	EDI-A-P-Index	SPAS-12	BIAQ-A
Body Dissatisfaction score					
Paper-based FRS	0.12 (0.25)	0.11 (0.30)	0.15 (0.16)	0.19 (0.07)	0.15 (0.15)
Allo. VR	0.09 (0.38)	0.10 (0.31)	0.13 (0.21)	0.15 (0.17)	0.23 (0.03 *)
Ego. VR	0.03 (0.79)	0.001 (0.99)	0.04 (0.67)	0.10 (0.371)	0.05 (0.63)
Allo. Mobile	−0.01 (0.93)	0.05 (0.66)	0.02 (0.82)	0.08 (0.45)	0.25 (0.02 *)
Body Distortion score					
Paper-based FRS	0.10 (0.36)	0.16 (0.12)	0.10 (0.34)	−0.03 (0.79)	0.16 (0.14)
Allo. VR	0.22 (0.04 *)	0.23 (0.03 *)	0.24 (0.02 *)	0.05 (0.66)	0.23 (0.03 *)
Ego. VR	0.06 (0.59)	0.07 (0.47)	0.15 (0.16)	−0.008 (0.94)	0.19 (0.08)
Allo. Mobile	0.28 (0.01 **)	0.30 (0.005 **)	0.13 (0.21)	−0.014 (0.90)	0.157 (0.16)

Note. EDI-A: Eating Disorder Inventory Scale; S-Index: Symptoms Index; P-Index: Personality; Trait Index SPAS-12: Social and Physique Anxiety Scale; BIAQ-A: Body Image Avoidance Questionnaire; Paper-based FRS: paper Figure Rating Scale; Allo. VR: Allocentric perspective from the *e*LoriCorps-IBRS 1.1; Ego VR: Egocentric perspective from the *e*LoriCorps-IBRS 1.1; Allo. Mobile: Allocentric perspective from the *e*LoriCorps-IBRS 1.1-Mobile. * *p* < 0.05; ** *p* < 0.01.

**Table 4:** Pearson’s correlations (with exact *p* values in brackets) for the subscales of questionnaires measuring components of BIDs and three rating scales administered to adolescents: a paper-based FRS, the *e*LoriCorps-IBRS 1.1 allocentric and egocentric perspectives, and the *e*LoriCorps-IBRS 1.1-Mobile allocentric perspective.

	Body Dissatisfaction				Body Distortion			
	Paper-FRS	Allo. VR	Ego. VR	Allo. M.	Paper-FRS	Allo. VR	Ego. VR	Allo. M.
EDI-A-BD	0.03 (0.82)	0.09 (0.39)	0.02 (0.89)	−0.05 (0.64)	0.11 (0.30)	0.22 (0.04 *)	0.09 (0.40)	0.21 (0.07)
EDI-A-DT	0.12 (0.26)	0.18 (0.09)	−0.02 (0.86)	0.13 (0.26)	0.18 (0.01)	0.23 (0.04 *)	0.09 (0.44)	0.17 (0.13)
EDI-A-BU	−0.17 (0.12)	−0.14 (0.21)	−0.09 (0.43)	0.11 (0.34)	0.01 (0.91)	−0.11 (0.32)	−0.001 (0.99)	−0.03 (0.77)
EDI-A-IN	−0.008 (0.94)	0.02 (0.84)	−0.08 (0.47)	0.03 (0.79)	0.16 (0.14)	0.19 (0.07)	0.18 (0.10)	0.24 (0.03 *)
EDI-A-PE	−0.02 (0.82)	0.001 (0.99)	0.003 (0.98)	0.07 (0.51)	0.08 (0.45)	0.11 (0.33)	0.10 (0.35)	−0.06 (0.62)
EDI-A-ID	0.001 (0.99)	0.11 (0.29)	0.19 (0.07)	−0.006 (0.96)	−0.03 (0.77)	0.06 (0.57)	0.002 (0.99)	−0.02 (0.85)
EDI-A-IA	−0.05 (0.65)	−0.02 (0.87)	−0.22 (0.04 *)	−0.05 (0.68)	−0.09 (0.42)	−0.02 (0.82)	−0.10 (0.35)	−0.04 (0.70)
EDI-A-MF	0.17 0.10	0.27 (0.01 *)	0.01 (0.90)	0.28 (0.01 *)	0.27 (0.01 *)	0.35 (0.001 **)	0.27 (0.01 *)	0.20 (0.08)
BIAQ-C	0.150 (0.17)	0.11 (0.30)	0.12 (0.25)	0.24 (0.03 *)	0.009 (0.93)	0.05 (0.64)	0.009 (0.93)	−0.04 (0.73)
BIAQ-S	0.11 (0.31)	0.20 (0.06)	−0.04 (0.69)	0.24 (0.03 *)	0.14 (0.21)	0.24 (0.02 *)	0.18 (0.09)	0.12 (0.28)
BIAQ-E	0.15 (0.16)	0.31 (0.003 **)	0.09 (0.36)	0.13 (0.25)	0.29 (0.007 **)	0.36 (0.001 **)	0.26 (0.01 *)	0.25 (0.02 *)
BIAQ-G	−0.03 (0.81)	−0.02 (0.82)	−0.11 (0.32)	−0.07 (0.52)	0.02 (0.84)	0.004 (0.97)	0.07 (0.55)	0.17 (0.12)

Note. EDI-A: Eating Disorder Inventory Scale; BD: Body Dissatisfaction Subscale; DT: Drive for Thinness Subscale; BU: Bulimia Subscale; IN: Infectiveness Subscale; PE: Perfectionism Subscale; ID: Interpersonal Distrust Subscale; IA: Interoceptive Awareness Subscale; MF: Maturity Fear Subscale; BIAQ-A: Body Image Avoidance Questionnaire; C: Clothing Subscale; S: Social Activity Subscale; E: Eating Restraint Subscale; G: Grooming and Weighing Subscale; Paper-FRS: paper Figure Rating Scale; Allo. VR: Allocentric perspective from the *e*LoriCorps-IBRS 1.1; Ego VR: Egocentric perspective from the *e*LoriCorps-IBRS 1.1; Allo. Mobile: Allocentric perspective from the *e*LoriCorps-IBRS 1.1-Mobile. * *p* < 0.05 ; ** *p* < 0.01.

## Data Availability

The data presented in this study are available on request from the corresponding author. The data are not publicly available due to ethical restrictions.
